# Convergence of genetic influences in comorbidity

**DOI:** 10.1186/1471-2105-13-S2-S8

**Published:** 2012-03-13

**Authors:** Richard C McEachin, Keerthi S Sannareddy, James D Cavalcoli, Alla Karnovsky, Jacqueline M Vink, Maureen A Sartor

**Affiliations:** 1 Center for Computational Medicine and Bioinformatics, University of Michigan, Ann Arbor, MI, 48105 USA; 2 Department of Biological Psychology, VU University, Van der Boechorststraat 1, 1081 BT Amsterdam, The Netherlands

## Abstract

**Background:**

Predisposition to complex diseases is explained in part by genetic variation, and complex diseases are frequently comorbid, consistent with pleiotropic genetic variation influencing comorbidity. Genome Wide Association (GWA) studies typically assess association between SNPs and a single-disease phenotype. Fisher meta-analysis combines evidence of association from single-disease GWA studies, assuming that each study is an independent test of the same hypothesis. The Rank Product (RP) method overcomes limitations posed by Fisher assumptions, though RP was not designed for GWA data.

**Methods:**

We modified RP to accommodate GWA data, and we call it modRP. Using p-values output from GWA studies, we aggregate evidence for association between SNPs and related phenotypes. To assess significance, RP randomly samples the observed ranks to develop the null distribution of the RP statistic, and then places the observed RPs into the null distribution. ModRP eliminates the effect of linkage disequilibrium and controls for differences in power at tested SNPs, to meet RP assumptions in application to GWA data.

**Results:**

After validating modRP based on both positive and negative control studies, we searched for pleiotropic influences on comorbid substance use disorders in a novel study, and found two SNPs to be significantly associated with comorbid cocaine, opium, and nicotine dependence. Placing these SNPs into biological context, we developed a protein network modeling the interaction of cocaine, nicotine, and opium with these variants.

**Conclusions:**

ModRP is a novel approach to identifying pleiotropic genetic influences on comorbid complex diseases. It can be used to assess association for related phenotypes where raw data is unavailable or inappropriate for analysis using other approaches. The method is conceptually simple and produces statistically significant, biologically relevant results.

## Background

Genome Wide Association (GWA) studies typically assess evidence of association between individual variants (e.g. SNPs) and a single-disease phenotype. Extending GWA to assess pleiotropic influences on comorbidity is a reasonable next-step in complex disease analysis. Approaches to GWA for comorbid phenotypes that combine raw data may not be possible if the raw data are unavailable or are inappropriate to combine (e.g., due to differences in data types or analytical methods). Fisher meta-analysis [1] combines the p-values from multiple studies and, if the individual GWA studies to be combined are independent and test the same hypothesis, the distribution of the Fisher statistic is chi-square. For comorbidity studies, we considered the related Rank Product (RP) approach introduced by Breitling, et al. [2]. RP combines data from multiple microarray studies, where samples may not be strictly independent and may test related hypotheses. Input to the RP method consists of a table with one column of probe identifiers, and one data column of ranks (1 to *N*) for each phenotype tested based on the fold-change values. The product of the ranks is calculated (RP statistic) for each row. To assess significance, the null distribution of the RP statistic is derived by randomly sampling from the ranks in each column, and forming many RP statistics. Each observed RP statistic is placed into the null distribution, and non-parametric p-values are calculated. We report here a modified RP method (modRP) that ensures that we meet the assumptions of RP in application to GWA by explicitly disrupting linkage disequilibrium (LD) and by grouping SNPs based on minor allele frequency (MAF) (see supplement for RP assumptions). In this work, we validated modRP based on available control studies, then found a novel, statistically significant, biologically relevant association between two SNPs and comorbid substance dependence phenotypes, providing a model for this gene-environment interaction and demonstrating the usefulness of the approach.

## Methods

For each analysis, we merged the datasets from individual studies by SNP identifier, ranked each column by p-value, calculated the observed RP for each SNP, and sorted SNPs by increasing RP. For each SNP, we downloaded chromosome position and MAF annotation from HapMart [3], or used annotation from the original study. To control for potential differences in power based on MAF, we grouped SNPs by low, medium, and high MAF (e.g., MAF < 10%, 10% < MAF < 25%, 25% < MAF < 50%). ModRP also uses SNP position to restrict random sampling to SNPs outside the potential range of LD. For Lind's data [4] we calculated correlations across the top 0.1% of SNPs (when ranked by RP), and across the complete dataset. For Yu's data [5], we calculated correlations across the top 0.5% of SNPs, and across the complete dataset. Details of the assumptions and control studies, as well as details of the modRP algorithm, are provided in the supplement.

We used 10^9 ^valid iterations (meeting both LD and MAF requirements) in permutation testing, performed tests in pairs, and ensured that each pair of tests yielded essentially the same results. If not, we increased the number of iterations until the criterion was met. We report the higher p-value for each pair of results. We applied a Bonferroni correction to adjust for multiple hypothesis testing, based on the number of SNPs. For comparison among methods, for each test we also performed traditional Fisher meta-analysis, modified Fisher (empirical p-values), and RP. See supplement for effectiveness of modRP and run time.

## Results

We first tested modRP using datasets from Lind, et al. [4], who combined alcohol dependence (AD) and nicotine dependence (ND) GWA datasets in a comorbidity study by developing a chi-square statistic, and applied it to two populations. This study found significant association with the comorbidity in one AD/ND population (positive control), and did not find evidence in a second AD/ND population (negative control). Lind's group reported significant association between three SNPs (rs7530302, rs1784300, rs12882384) and comorbid AD/ND in the Australian population (Table 1). For these SNPS, results based on modRP are very similar to those derived by Lind. All three SNPS are significantly associated with the comorbid phenotype, though modRP is slightly more conservative than Lind's approach. For rs7530302 and rs12882384, a) the Fisher test result varies from both Lind's and modRP result, b) the mod-Fisher result varies from Lind's values, Fisher, and modRP and c) RP differs from all of the other results. This effect is not seen for rs1784300, where all five methods yield a similar level of significance. In the combined Australian/Dutch populations we did not find significant association between any SNP and AD or ND using any of the test methods, consistent with Lind's results (Table 1). We then applied modRP to datasets developed by Yu, et al. [5], who performed meta-analyses on four single-disease phenotypes (cocaine, opium, nicotine, and alcohol dependence), in a combined population based on African American (AA) and European American (EA) sub-populations. In replicating Yu's meta-analyses, modRP does not find any significant SNPs, consistent with the other four methods.

**Table 1:** Comparison of modRP results to control studies

Lind, et al., Published Results			Our Results, Corrected Threshold: 6.54E-08				
**SNP**	**p-value**		**SNP**	**Fisher**	**Mod Fisher**	**RP**	**Mod RP**

		***Australian (Pos Control)***					

rs7530302	**1.90E-09**	**AD/ND Comorbidity**	rs7530302	**5.29E-08**	2.33E-07	**5.60E-08**	**3.39E-08**

rs1784300	**2.60E-09**		rs1784300	**1.19E-09**	**2.00E-09**	**6.00E-09**	**3.57E-09**

rs12882384	**4.86E-08**		rs12882384	2.16E-07	5.51E-07	1.08E-07	**5.72E-08**

		***Aus/Dutch (Neg Control)***					

rs2247219	6.23E-06	**AD/AD/AD Meta**	rs2247219	2.22E-05	2.02E-05	1.52E-05	1.41E-05

rs6705087	4.80E-06	**ND/ND Meta**	rs6705087	1.16E-05	1.12E-05	4.02E-06	5.64E-06

	N/A	**AD/ND/AD/AD/ND Co**	rs1784300	**3.31E-08**	**2.20E-08**	1.05E-07	1.27E-07

**Yu, et al., Published Results**			**Our Results, corrected Threshold: 8.88E-06**				

**SNP**	**p-value**	**Single Phenotypes (Neg)**	**SNP**	**Fisher**	**Mod Fisher**	**RP**	**Mod RP**

rs1938584	3.00E-03	**Cocaine**	rs1476880	1.36E-04	1.41E-04	1.32E-04	1.41E-04

rs1516003	9.00E-04	**Opium**	rs1333962	1.80E-02	1.16E-05	1.01E-05	1.15E-05

rs769592	2.00E-03	**Nicotine**	rs2194	4.20E-02	3.48E-04	3.47E-04	3.47E-04

rs1937443	4.00E-03	**Alcohol**	rs6505380	1.30E-05	1.37E-04	1.48E-04	1.37E-04

Comparison of Lind and Yu results with results from our four tested methods. Tests that satisfied the Bonferroni correction threshold are in bold.

In a novel study (Table 2) we assessed evidence for pleiotropy in four comorbidities, in AA and EA populations plus the combined population, based on single-disease p-values output from Yu's study [5]. In each case, we checked the four-way (cocaine/opium/nicotine/alcohol), three-way (e.g., cocaine/opium/nicotine), and two-way (e.g., cocaine/opium) comorbidities. In the AA population, we found rs1426165 to be significantly associated with cocaine/nicotine dependence comorbidity, with a p-value of 3.62E-06. This SNP is in the coding region of the *ADAMTSL3 *gene (ADAMTS-like 3, a disintegrin-like and metalloprotease domain with thrombospondin type I motifs-like 3, Entrez GeneID 57188). In the EA population, we found rs1476880 to be significantly associated with cocaine/nicotine comorbidity, with a p-value of 5.38E-06. This SNP tags the *SOD3 *gene (superoxide dismutase 3, Entrez GeneID 20657). In addition, evidence for association of rs1476880 with the three-way comorbidity of cocaine/opium/nicotine dependence is even more significant (p-value 2.26E-06), consistent with an amplified signal in the three-way comorbidity, and in the combined population. A systems biology interpretation of these results is provided in the supplement, using GeneGo's MetaCore software (GeneGo Inc., St. Joseph, MI).

**Table 2:** Results from modRP analysis of cocaine (C), opium (O), nicotine (N), and alcohol (A) dependence comorbidities in Combined, EA, and AA populations.

COMBINED (EA & AA) Population									
**Four**			**Three**			**Two**			

						CO	rs1476880*	3.30E-05	

**CONA**	rs1476880	1.55E-05	CON	rs1476880*	**2.26E-06**	CN	rs1476880*	**5.30E-06**	**SOD3**

**CONA**	rs1426165	2.68E-03	CNA	rs1426165	6.87E-04	CN	rs1426165	2.97E-04	ADAMTSL3

**CONA**	rs1169791*	1.23E-05	CNA	rs1925156*	1.20E-05	CA	rs1938584*	3.07E-05	

			COA	rs1938584*	3.35E-05	ON	rs1860148*	5.61E-05	

			ONA	rs1385513*	1.07E-04	OA	rs726427*	3.66E-04	

						NA	rs2169325*	1.57E-04	

**EUROPEAN AMERICANS**									

**Four**			**Three**			**Two**			

						CO	rs1476880	3.32E-05	

**CONA**	rs1476880	1.59E-05	CON	rs1476880*	**2.23E-06**	CN	rs1476880*	**5.38E-06**	**SOD3**

**CONA**	rs1426165	2.67E-03	CNA	rs1426165	6.87E-04	CN	rs1426165	2.97E-04	ADAMTSL3

**CONA**	rs1169791*	1.26E-05	CNA	rs1925156*	1.20E-05	CA	rs1938584*	3.06E-05	

			COA	rs1938584*	3.33E-05	ON	rs1860148*	5.61E-05	

			ONA	rs1385513*	1.07E-04	OA	rs726427*	3.66E-04	

						NA	rs2169325*	1.57E-04	

**AFRICAN AMERICANS**									

**Four**			**Three**			**Two**			

			CON	rs726344*	2.70E-05	CO	rs1015343*	9.50E-05	

**CONA**	rs1476880	2.46E-04	CON	rs1476880	4.47E-05	CN	rs1476880	2.01E-05	SOD3

**CONA**	rs1426165	7.44E-05	CNA	rs1426165*	**1.73E-05**	CN	rs1426165*	**3.62E-06**	**ADAMTSL3**

			COA	rs910633*	8.32E-05	CA	rs763869*	4.54E-05	

						ON	rs897119*	1.05E-04	

**CONA**	rs795734*	5.03E-05	ONA	rs795734*	9.12E-06	OA	rs795734	7.06E-05	

						NA	rs795734	1.07E-04	

In each population, the 4-way (Four), 3-way (Three), and 2-way (Two) comorbidities proceed from left to right. * Indicates the most significant result for each comorbid phenotype, in each population. For reference, complete results for rs1426165 and rs1476880 are included in each population set. Bonferroni Corrected Threshold: 8.88e-006

## Discussion

In this work, we introduce modRP, a method to identify pleiotropic influences on comorbid phenotypes, and compare modRP to four related methods. ModRP combines summary data from related GWA studies, while controlling for minor allele frequency and linkage disequilibrium. Comparison of modRP performance to studies by Lind, et al., [4] and Yu, et al.,[5] showed that modRP produces results consistent with available positive and negative control studies. While no one knows the "true" genetic influences in these populations, these comparisons provide evidence of modRP's effectiveness in field studies. In the test study, association of SNP rs1426165 with the cocaine/opium/nicotine comorbidity highlights the well-developed body of evidence for the influence of oxidative stress in substance dependence. Superoxide dismutases catalyze the dismutation of two superoxide radicals into hydrogen peroxide and oxygen and protect tissues from oxidative stress. *SOD3 *has not been previously associated with drug abuse, though there are documented connections between oxidative stress and nicotine [6], heroin [7], and cocaine [8] dependence. It has been suggested that oxidative mechanisms mediate the processes of drug addiction and toxicity [9,10] and that antioxidants may have therapeutic potential in managing these conditions. Little has been published on *ADAMTSL3*, although the ADAM gene family has been associated with multiple diseases induced by oxidative stress [11,12].

## Conclusions

ModRP combines prior evidence of association with related phenotypes to identify novel variants which may influence comorbid phenotypes through common underlying mechanisms. The algorithm uses p-values for association with single-disease phenotypes as input, combines this evidence to form a test statistic for each SNP, and assesses the significance of each test statistic. Raw data, which may be unavailable or inappropriate for combining, is not required by modRP. The algorithm provides significant insight into genetic variation influencing pleiotropy. This work opens the door to analysis of comorbid or single-disease phenotypes, assessed in a single population or in independent populations.

## Competing interests

The authors declare that they have no competing interests.

## Authors' contributions

RCM, MAS, JDC, and KSS conceived the approach, developed and tested modRP, and drafted the paper. AK and JMV interpreted biological significance.

## Supplementary Material

Additional file 1 **Details of the algorithm, control studies and systems biology**. A Word document providing additional details on assumptions, studies used as controls, and systems biology interpretation. Click here for file
