# Supplementary material for: Convergence of genetic influences in comorbidity
Source: BMC Bioinformatics. 2012 Mar 13;13(Suppl 2):S8. doi: 10.1186/1471-2105-13-S2-S8 (PMC3375629; doi:10.1186/1471-2105-13-S2-S8)
Supplement: Additional file 1 — Details of the algorithm, control studies and systems biology. A Word document providing additional details on assumptions, studies used as controls, and systems biology interpretation. [file 1471-2105-13-S2-S8-S1.pdf]

## Additional Background

Individuals are predisposed to complex diseases by both genetic variation and environmental influences [1] and they are frequently comorbid, such that individuals with a certain complex disease may be at elevated risk for other complex diseases. For example, patients with bipolar disorder are at risk for substance use disorders [2], patients with diabetes are at risk for hypertension [3], and alcohol dependent individuals show high rates of nicotine dependence[4]. In all of these cases, we have evidence that the comorbid diseases are both influenced by genetic variation [1, 5, 6], and the population is over-represented for the comorbidity [2, 3], consistent with the existence of one or more pleiotropic genetic influences common to both diseases.

One approach to GWA in comorbidity defines participants in a single study as being affected if they exhibit both disease phenotypes and as unaffected if they exhibit neither disease phenotype. This approach may be effective [4], though it limits sample size because many potential study participants are expected to have one or the other of the disease phenotypes. Since these individuals would be neither affected nor unaffected, they would not be included in the analysis. Another approach is to combine raw data from studies on related phenotypes, then calculate the appropriate association statistics [7, 8]. This can create a problem if the phenotype definitions are not identical. For example, one study may consider participants as being affected using a restrictive diagnosis of bipolar I disorder, while another study may consider participants as being affected based on a less restrictive diagnosis of either bipolar I and bipolar II disorder, a related but not identical phenotype. This problem also presents itself when applying Fisher meta-analysis [9], which combines p-values from studies that are independent tests of the same hypothesis. Also, with respect

to independence, multiple longitudinal studies are ongoing (e.g. Women's Health Initiative, Framingham) and in each study, data are being gathered on multiple phenotypes. Results of single disease GWA studies have shown promise in these applications [10] but comorbidities are expected to be found in each of these populations [11, 12], as they are in the general population. Since multiple phenotypes are being assessed in a single population, using standard Fisher meta-analysis would violate the assumption of independence.

# Assumptions

### The Rank Product (RP) and modified RP (modRP) methods

According to Breitling, et al., derivation of the null distribution of RP statistics depends on relatively mild assumptions [13]: "(1) that relevant expression changes affect only a minority of genes, (2) measurements are independent between replicate arrays, (3) most changes are independent of each other, and (4) measurement variance is about equal for all genes." Our modifications account for the aspects of GWA studies that do not meet the above assumptions. Translating Breitling's assumptions for application of the RP method to GWA, we assume: (1) a minority of SNPs are associated with the comorbid phenotype, (2) association evidence is independent between phenotypes, (3) most association evidence is independent between SNPs, and (4) measurement variance is about equal for all SNPs. With respect to (1), prior to running modRP, we check for a concentration of association evidence in the top $N$ SNPs, where $N$ is determined by the size of the input dataset. If a minority of SNPs are associated with the comorbid phenotype, they should be at the top of the list and their ranks should be similar (all relatively low), so we expect to see higher correlation in ranks for the SNPs at the top of the list than at the bottom of the list. With respect to assumption (2), under the null assumption of no association the observed ranks are random, so the association measures are independent across

phenotypes. We test this assumption prior to running modRP by looking for correlation across phenotypes. If there is significant association between one or more SNPs and the comorbid phenotype, correlation between columns will be greater than zero, though we expect the value to be small. With respect to assumption (3), under the same null assumption of no association the observed evidence for association at each SNP is random, so each SNP is independent. However, since linkage disequilibrium (LD) between SNPs may invalidate this assumption, we explicitly disrupt LD in modRP. With respect to (4), SNP minor allele frequencies (MAFs) influence power in association testing, so variance in the signal strength among SNPs could depend on MAF. We explicitly account for this possibility by grouping SNPs based on MAF.

# Control studies

### Lind data

Lind, et al., [4] performed a GWA analysis for AD, another for ND, and a third for the comorbidity, in an Australian population. They replicated the AD and ND steps in a Dutch population, and then performed analyses for AD, ND, and AD/ND on the combined studies for the top 10,000 SNPs. Notably, the Australian DNA samples were pooled, so this is an example of a study where combining individuals' genotype and phenotype data with another study is not practical. Also note that the Dutch study on AD used two diagnostic criteria: DSM IV based lifetime CIDI interviews in the NESDA [4] study and CAGE [4, 14] in the NTR study. We prepared each of these datasets for input to modRP, selecting the 764,218 SNPs that matched in the two studies, and replicated the previous analyses. To assess the importance of meeting the assumptions required by Fisher meta-analysis, for each study we performed a traditional Fisher meta-analysis, assuming a chi-square distribution for the Fisher statistic, as well as a modified Fisher meta-analysis, where we developed the null distribution of the Fisher statistic via

sampling. To assess the importance of meeting the assumptions for RP, for each study we performed a standard RP in addition to the modified RP. We then compared results across the four methods (Fisher, mod Fisher, RP, and modRP).

**Correlations**: We tested correlations with respect to assumptions (1) and (2), Table S1. In both the Lind and Yu datasets, correlations among the top N SNPs ("Top 0.1%" for Lind's data and "Top 0.5%" for Yu's data) are consistently higher than in the complete datasets "All". This result is consistent with assumption (1). Also, the magnitude of correlation across phenotypes in the complete datasets is small (<11%), consistent with assumption (2).

| **Lind, et al.** | | | | |
|---|---|---|---|---|
| **Top 0.1%** | **Aus AD** | **Aus ND** | **Dutch AD1** | **Dutch AD2** |
| **Aus ND** | 6.3% | | | |
| **Dutch AD1** | 12.9% | 8.5% | | |
| **Dutch AD2** | 14.4% | 15.6% | 3.6% | |
| **Dutch ND** | 18.3% | 18.2% | 4.9% | 7.7% |
| **All** | **Aus AD** | **Aus ND** | **Dutch AD1** | **Dutch AD2** |
| **Aus ND** | 2.0% | | | |
| **Dutch AD1** | 0.0% | 0.2% | | |
| **Dutch AD2** | 0.0% | 0.0% | 0.4% | |
| **Dutch ND** | 0.0% | 0.0% | 0.4% | 0.2% |
| **Yu, et al.** | | | | |
| **Top 0.5%** | **Cocaine** | **Opium** | **Nicotine** | |
| **Opium** | 26.6% | | | |
| **Nicotine** | 18.9% | 15.7% | | |
| **Alcohol** | 16.5% | 27.8% | 18.6% | |
| **All** | **Cocaine** | **Opium** | **Nicotine** | |
| **Opium** | 5.4% | | | |
| **Nicotine** | 10.5% | 4.4% | | |
| **Alcohol** | 6.5% | 2.6% | 7.9% | |

Table S1: **Magnitudes of Correlations, Top n% SNPs versus Total.** Comparison of magnitudes of correlations between ranks for Lind data (Alcohol, Nicotine Dependence) and Yu data (Cocaine, Opium, Nicotine, and Alcohol Dependence). Each phenotype is compared to all other phenotypes in the group. For each group, the "Top n%" (by Rank Product statistic) were correlated separately, then the entire set "All" were correlated. In both cases, the Top n% group shows a concentration of association evidence, while the overall correlation is modest.

### Yu data

Yu, et al., [15] performed GWA analysis on six substance-use phenotypes (cocaine, opium, and alcohol dependence, as well as two measures of nicotine dependence and cocaine induced paranoia), in two study populations (AA, EA) plus the combined population. Yu, et al., used 5633 “tagging” SNPs across the autosomal genome, spaced on average 518 kb apart. Tagging SNPs are selected to survey potential causal variants in LD with the tagging SNPs. We prepared the Yu datasets for input and used modRP on the cocaine, opium, and alcohol dependence datasets, as well as the nicotine dependence dataset based on DSM IIIR diagnostic criteria. We first used RP to replicate the meta-analyses that Yu performed on each of the single disease phenotypes. Where they combined the EA and AA populations, we used modRP to combine summary statistics from the two studies. For each population, we then looked at each of the 4-way, 3-way, and 2-way comorbidities. We adopted the same MAF values that Yu used, for each population.

## ModRP algorithm

The modRP algorithm is implemented as a Perl script, available for research purposes from mceachin@umich.edu or by download from www.ncibi.org :

1. Import the observed data, annotation, and parameter settings via the input file
   A. Select “m” (between 1 and N), as the number of SNPs to output with association statistics
   B. Select the number of iterations to be completed
   C. Select the minimum distance between SNPs expected to disrupt LD
2. Store ranks, RP, and annotation data on all SNPs in an array
3. Develop the null distribution of the RP statistic:

A. Randomly select one rank statistic from each column (phenotype) and confirm that the SNPs related to the selected ranks are not linked; ensure all SNPs are on different chromosomes or, if any two are on the same chromosome, confirm that they are at least the user-selected distance apart
B. Confirm that the SNPS related to the selected ranks are all in the same MAF group
C. if A and B are true, form one RP for the null distribution by multiplying the selected ranks
    i. else - go to A
D. If the null RP is smaller than each of the smallest “m” observed RPs, increment the variable that counts null RP values smaller than the observed RP (p-value count)
E. Iterate over the user-selected number of cycles

3. Calculate p-values for the SNPs with the smallest “m” RPs, as the proportion of null RPs equal to or less than the observed RP
    i. (p-value count + 1) / (#iterations +1)
    ii. Apply a Bonferroni correction based on the number of SNPs tested
4. Output p-values for SNPs with the smallest “m” RPs and annotate SNPs that meet the Bonferroni correction criteria as “significant’ in the output
5. Repeat the test in its entirety
    i. Confirm that the solution is essentially the same
    ii. Else, go to 1 and increase the number of iterations

## Systems biology

Given two candidate genes derived from analysis of the Yu dataset, we placed them into biological context using the MetaCore database provided by GeneGo (GeneGo Inc., St. Joseph, MI). We input our candidate genes and used MetaCore's "build network" algorithm, with the following options: shortest paths, merged network, use canonical pathways, maximum steps = 4, show disconnected seed nodes, show shortest path edges only, discard low trust interactions, use functional and binding interactions, and use all compound-target interactions. After adding cocaine and nicotine to the network, we trimmed off all paths longer than two steps, to improve the clarity of the figure. GeneGo does not provide opium as a metabolite, so we added the opium receptors to the network, and again trimmed the paths longer than two steps to improve clarity.

To place the genes tagged by these two variants into biological context, we first used GeneGo's MetaCore software [16] to develop a network connecting the *SOD3* and *ADAMTSL3* (Figure S1). Notably, the genes are connected by a relatively simple path: *SOD3* activates transcription factor HIF1A, which activates transcription of signaling protein IFN-gamma, which inhibits expression of Calpain3, which cleaves *ADAMTSL3*. This path fits into a larger network that models these genes' roles in susceptibility to both cocaine and nicotine dependence, consistent with our modRP results. Figure S2 shows the multiple simple paths from nicotine and cocaine to the genes in the path between *SOD3* and *ADAMTSL3*. Clearly, both cocaine and nicotine are likely to exert environmental influences on HIF1A and IFN-gamma, and it is reasonable to expect that variation in either *SOD3* or *ADAMTSL3* would interact with these environmental effects. The Gene Ontology biological process "regulation of monooxygenase activity" is the most significantly over-represented among genes in this pathway (p-value 7.70E-11).

ModRP results suggest that *SOD3* is associated with cocaine/nicotine dependence, as well as opium dependence. Figure S3 illustrates the influence of opium on this network. Notably, opium is more closely associated with *SOD3* than *ADAMTSL3* in this network, consistent with the evidence derived from the comorbidity study. Also consistent with comorbidity of psychiatric and substance use disorders, this network is strongly associated with “Depressive Disorder, Major” (p-value 3.43E-14) and “Substance-Related Disorders” (p-value 7.71e-12). Interestingly, this network is significantly associated with “Neoplasms, Squamous Cell” (p-value 1.79E-11) and several other cancers, as well as “Respiration Disorders” (p-value 4.23E-11) and “Muscular Diseases” (p-value 6.58E-11), consistent with a broadened impact of genetic variation associated with psychiatric and substance use disorders, on other medical phenotypes. This is an effect that we have seen in our recent work [17-19]. In addition, eight of the genes in this network are known targets of therapeutic drugs. Arguably, any of the drugs in this set could have therapeutic effects in treatment for cocaine, opium, and/or nicotine addiction.

## Effectiveness of modRP

We consider several factors that may influence the effectiveness of modRP. The algorithm depends on previously developed summary data. This is a plus, in that the data may be readily available, though care should be taken to assess characteristics or limitations that are not evident in a simple table of SNPs with p-values. For example, while modRP is suited to assessing comorbid phenotypes, the phenotypes assessed in each study must be well defined or the comorbid phenotype will not be well defined. In general, this information should be available in the manuscript describing the work. Also, the strength of the association signal seen in any particular study depends on the true effect size and strength as well as experimental factors such as sample size, genotyping platform, and phenotyping

effectiveness. Notably, in the Lind study, the data from the Dutch group was imputed so we were able to match virtually all of the SNPs used in the Australian study. We foresee the possibility that data will come from studies where researchers are using SNP arrays to look at copy number variations in cancers. These studies may be suited to modRP because, though the sample sizes may be small, the effect sizes may be much larger. Equally, especially in cancer studies, modRP may be appropriate for combining haplotype analyses.

## Run time

By using sampling to develop the null distributions of the RP statistics, we do not depend on distributional assumptions.  However, sampling requires high-performance computing capabilities and additional time. In general, we used modRP to assess empirical p-values for a subset of SNPs that are most likely to show significant association (top “m” SNPs, ranked by RP statistic). To assess the top 10 SNPs for either the Lind data or the Yu data, run time for $10^9$ iterations on an Intel Core i7 CPU, Q 720 @1.60GHz processor, with 64 bit Windows OS took two to three hours, depending on the options. Larger input datasets do not appreciably increase run time, but assessing more SNPs for association and performing more iterations both increase run-time.

## Additional file 1, figure legends

#### Figure S1  - Pathway connecting SOD3 with ADAMTSL3

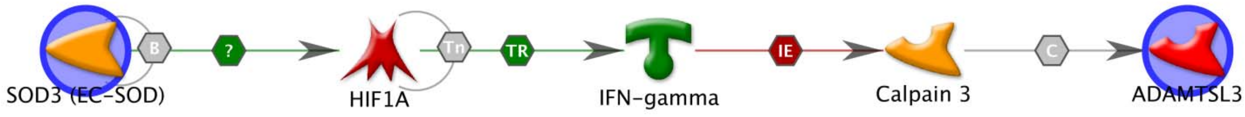


SOD3 activates HIF1A, which activates transcription of IFN-gamma, which represses expressin of Calpain 3, which cleaves ADAMTSL3.

**Figure S2 - Cocaine and nicotine interact with SOD3 with ADAMTSL3**

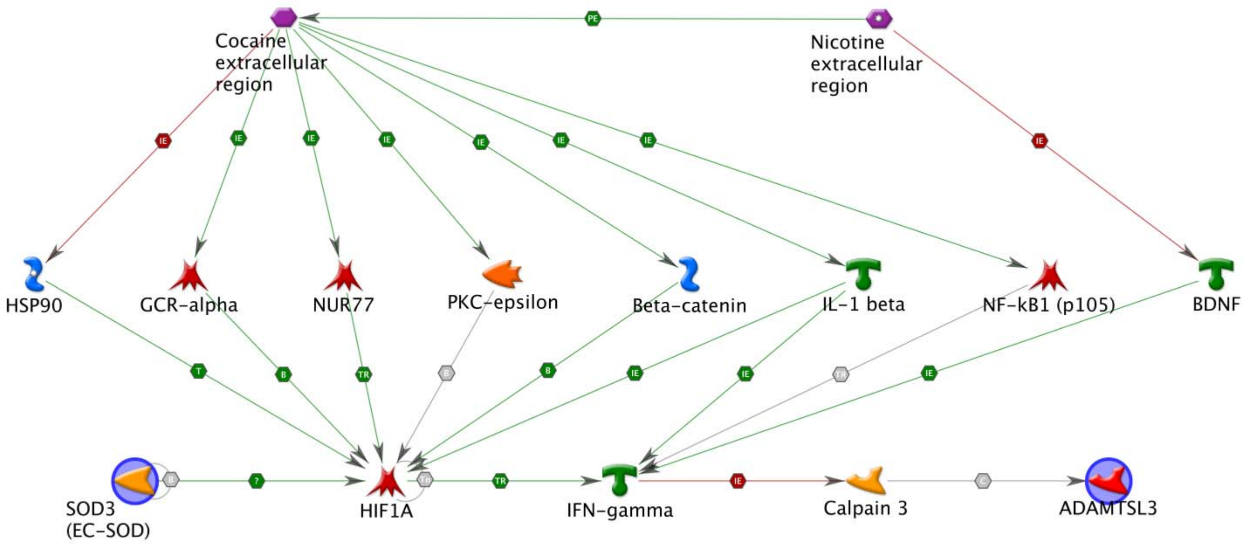


Both cocaine and nicotine act on the SOD3/ADAMTSL3 pathway via multiple intermediaries.

**Figure S3 - Opium also interacts with SOD3 with ADAMTSL3**

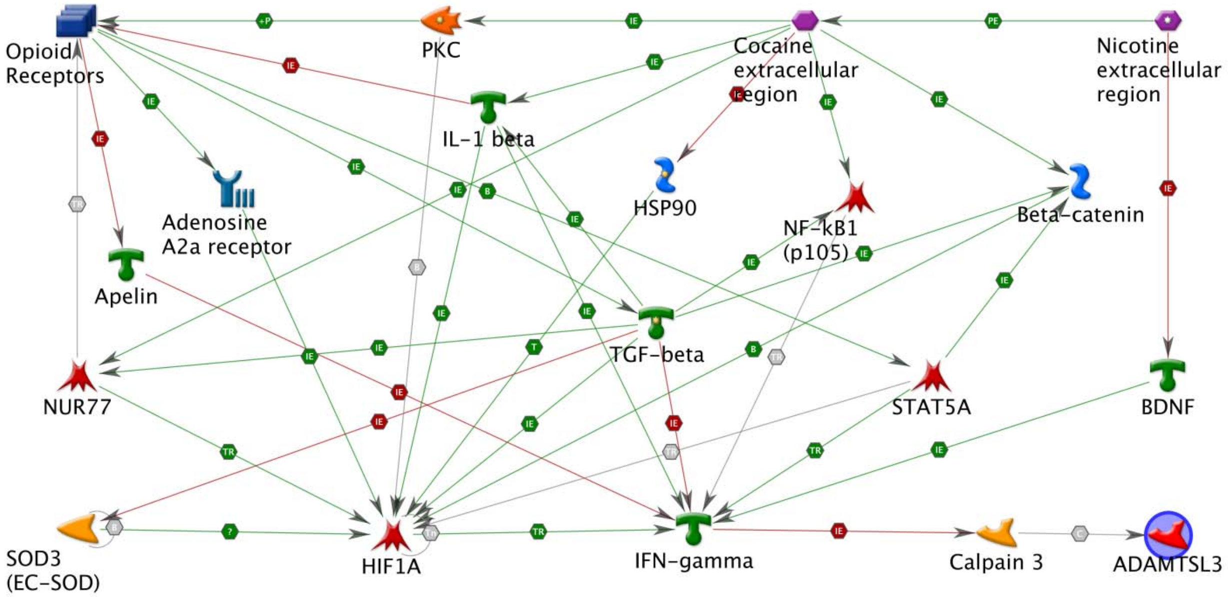


As with cocaine and nicotine, opium acts on the SOD3/ADAMTSL3 pathway via multiple intermediaries.

## Additional file 1, References


1. Hirschhorn JN, Gajdos ZK: **Genome-wide association studies: results from the first few years and potential implications for clinical medicine**. *Annu Rev Med* 2011, **62**:11-24.
2. Levin FR, Hennessy G: **Bipolar disorder and substance abuse**. *Biol Psychiatry* 2004, **56**(10):738-748.
3. Mooradian AD: **Cardiovascular disease in type 2 diabetes mellitus: current management guidelines**. *Arch Intern Med* 2003, **163**(1):33-40.
4. Lind PA, Macgregor S, Vink JM, Pergadia ML, Hansell NK, de Moor MH, Smit AB, Hottenga JJ, Richter MM, Heath AC *et al*: **A genomewide association study of nicotine and alcohol dependence in Australian and Dutch populations**. *Twin Res Hum Genet* 2010, **13**(1):10-29.
5. Nothen MM, Nieratschker V, Cichon S, Rietschel M: **New findings in the genetics of major psychoses**. *Dialogues Clin Neurosci* 2010, **12**(1):85-93.
6. Bierut LJ: **Genetic vulnerability and susceptibility to substance dependence**. *Neuron* 2011, **69**(4):618-627.
7. Bolormaa S, Pryce JE, Hayes BJ, Goddard ME: **Multivariate analysis of a genome-wide association study in dairy cattle**. *J Dairy Sci* 2010, **93**(8):3818-3833.
8. Liu YZ, Pei YF, Liu JF, Yang F, Guo Y, Zhang L, Liu XG, Yan H, Wang L, Zhang YP *et al*: **Powerful bivariate genome-wide association analyses suggest the SOX6 gene influencing both obesity and osteoporosis phenotypes in males**. *PLoS One* 2009, **4**(8):e6827.

9. Fisher RA: **Combining Independent Tests of Significance**. *The American Statistician* 1948, **2**:30.

10. WTCCC: **Genome-wide association study of 14,000 cases of seven common diseases and 3,000 shared controls**. *Nature* 2007, **447**(7145):661-678.

11. Fox CS: **Cardiovascular disease risk factors, type 2 diabetes mellitus, and the Framingham Heart Study**. *Trends Cardiovasc Med* 2010, **20**(3):90-95.

12. Brennan-Calanan RM, Genco RJ, Wilding GE, Hovey KM, Trevisan M, Wactawski-Wende J: **Osteoporosis and oral infection: independent risk factors for oral bone loss**. *J Dent Res* 2008, **87**(4):323-327.

13. Breitling R, Armengaud P, Amtmann A, Herzyk P: **Rank products: a simple, yet powerful, new method to detect differentially regulated genes in replicated microarray experiments**. *FEBS Lett* 2004, **573**(1-3):83-92.

14. Ewing JA: **Detecting alcoholism. The CAGE questionnaire**. *JAMA* 1984, **252**(14):1905-1907.

15. Yu Y, Kranzler HR, Panhuysen C, Weiss RD, Poling J, Farrer LA, Gelernter J: **Substance dependence low-density whole genome association study in two distinct American populations**. *Hum Genet* 2008, **123**(5):495-506.

16. Nikolsky Y, Ekins S, Nikolskaya T, Bugrim A: **A novel method for generation of signature networks as biomarkers from complex high throughput data**. *Toxicol Lett* 2005, **158**(1):20-29.

17. McEachin RC, Chen H, Sartor MA, Saccone SF, Keller BJ, Prossin AR, Cavalcoli JD, McInnis MG: **A genetic network model of cellular responses to lithium treatment and cocaine abuse in bipolar disorder**. *BMC Syst Biol* 2010, **4**:158.

18. McEachin RC, Saccone NL, Saccone SF, Kleyman-Smith YD, Kar T, Kare RK, Ade AS, Sartor MA, Cavalcoli JD, McInnis MG: **Modeling complex genetic and environmental influences on comorbid bipolar disorder with tobacco use disorder**. *BMC Med Genet* 2010, **11**:14.
19. McEachin RC, Keller BJ, Saunders EF, McInnis MG: **Modeling gene-by-environment interaction in comorbid depression with alcohol use disorders via an integrated bioinformatics approach**. *BioData Min* 2008, **1**(1):2.
